# Exploring graphical approaches to assess the impact of an additional trial on a decision model via updated meta-analysis

**DOI:** 10.1017/rsm.2025.10011

**Published:** 2025-06-04

**Authors:** Will Robinson, Alex Sutton, Clareece Nevill, Nicola Cooper

**Affiliations:** Biostatistics Research Group, Department of Population Health Sciences, University of Leicester, Leicester, UK

**Keywords:** economic decision modelling, evidence-based research, funnel plot, meta-analysis, statistical methodology, study design

## Abstract

Graphical displays are often utilised for high-quality reporting of meta-analyses. Previous work has presented augmentations to funnel plots that assess the impact that an additional trial would have on an existing meta-analysis. However, decision-makers, such as the National Institute for Health and Care Excellence in the United Kingdom, assess health technologies based on their cost-effectiveness, as opposed to efficacy alone. Motivated by this fact, this article outlines a novel approach, developed for augmenting funnel plots, based on the ability of an additional trial to change a decision regarding the optimal intervention. The approach is presented for a generalised class of economic decision models, where the clinical effectiveness of the health technology of interest is informed by a meta-analysis, and is illustrated with an example application. The ‘decision contours’ produced from the proposed methods have various potential uses not only for decision-makers and research funders but also for other researchers, such as meta-analysts and primary researchers designing new studies, as well as those developing health technologies, such as pharmaceutical companies. The relationship between the new approach and existing methods for determining sample size calculations for future trials is also considered.

## Highlights

### What is already known?

- Previously described statistical significance contours were a useful foundation for allowing meta-analysts to visually assess how robust existing meta-analyses are to new evidence changing the conclusions regarding a treatment’s effectiveness, and for basing evidence-based sample size calculations for future trials on.

### What is new?

- This approach has been extended here to consider the cost-effectiveness of the intervention, via the impact on a decision model, which is often of primary interest to regulators and decision-makers. In addition to providing boundaries for where the treatment decision changes, the plots presented indicate the impact of future trials (via simulation), potentially informing future sample size calculations.

### Potential impact for RSM readers

- The methods described are potentially useful for those conducting evidence synthesis, informing economic decision models, and/or those considering the design of related future trials.

## 1 Introduction

Meta-analysis is a standard statistical methodology for combining relevant quantitative information from multiple studies and is used to answer many health research questions. Graphical displays are heavily utilised in the reporting of meta-analysis findings, as well as for the assessment of characteristics such as heterogeneity and publication bias.^1^^,^ ^2^

Langan et al.^3^ proposed augmentations to one of the most used graphical displays in meta-analysis, the funnel plot, which has previously been used to assess publication bias. These were contours that overlay the funnel plot and highlight the impact that a single additional trial could have on the results and other features of an existing meta-analysis; in particular, the statistical significance of the estimate for a health intervention’s efficacy. These were suggested to be useful for various purposes, including establishing the current robustness of a meta-analysis, informing sample-size calculations for future trials that may be added to the meta-analysis,^4^ and the prioritisation of the updating of meta-analyses when choosing from a portfolio of many,^5^ which has been shown not to be a simple task.^6^

The primary focus of this article is informing the sample size of future randomised controlled trials (RCTs), although the methods proposed could be used in any of the aforementioned situations.

Currently, sample size calculations are most commonly conducted/justified through ensuring acceptable power (e.g., 80%) under a frequentist statistical hypothesis testing paradigm. However, since meta-analyses of well-conducted RCTs are at the top of established hierarchies of effectiveness evidence, an argument can be made that it is the subsequent meta-analysis, when updated to include the new trial, that will be most impactful for policymaking and not the new trial results on their own. Previous work has described an approach to calculate sample size based on this perspective, when considering statistical significance^4^ or clinically important treatment differences,^7^ and the augmentations to the funnel plot by Langan et al. facilitate a visual exploration of this and provide insight into the calculations.^3^ This work endeavoured to make sample size calculations more evidence-based; however, it is not the only alternative approach to sample size calculations. Notably, the expected value of sample information (EVSI) has been used to derive sample size calculations and justify new trials using a fully rational economic decision theoretical framework.^8^ The EVSI method considers the impact a new trial will have on the cost-effectiveness of a new treatment by assessing the likely impact of the trial on an economic decision model. Such calculations are a radical departure from traditional sample size calculations based on frequentist hypothesis testing since they necessarily consider the costs and health benefits of the new trial and determine the sample size by maximising the difference between the two. The required calculations are also more complex due to (i) the need to reflect lifetime costs and health benefits of alternative treatments based on all relevant evidence, and (ii) the intensive simulation process required to reflect the potential impact of alternative sample sizes on decision uncertainty. Hence, such methods can be seen as a more rational approach than justifying a trial sample size on criteria such as an arbitrary level of statistical significance. This is also consistent with the fact that when health technologies are evaluated by decision-makers such as The National Institute for Health and Care Excellence (NICE), they are concerned with the cost-effectiveness of that technology and look beyond its efficacy.^9^

The meta-analysis-based methods proposed by Sutton and others (as described earlier)^4^^,^ ^7^ can be seen as a ‘halfway house’ between traditional sample size calculations and the use of EVSI methods. Both these methods consider the impact of the new trial on the totality of the clinical effectiveness evidence via meta-analysis. However, several further quantities are required for EVSI such as those related to: (i) the costs of the intervention; (ii) multiple clinical outcomes (including side effects) that may be pertinent to a treatment decision; and (iii) the cost and health impact of the new trial.

This article extends the ‘statistical significance contours’ plot ideas proposed by Langan et al.,^3^ by considering the impact that an additional trial in a meta-analysis has on an economic decision model, informed by the meta-analysis. However, unlike EVSI, it does not consider the cost of the new trial nor the expected benefit the trial will generate since it focuses on changing the outcome of the decision model (and the uncertainty around this decision) rather than the full (economic) implications of such a change. The plots proposed here have been developed to assist with ascertaining the likely impact the new evidence on effectiveness (as generated from an RCT) will have on a decision derived from a decision model, as well as the uncertainty associated with that decision. More specifically, such plots could be used to justify, or provide insight for, the sample size for a future trial by either using them on their own or in conjunction with one of the other sample size calculation approaches, including those considered above. We further consider the relative merits of the approach compared to the alternatives in the discussion.

The article first introduces all the necessary concepts; specifically, Section 2 describes the underlying meta-analytic methods relevant to the developments of this article, introducing the funnel plot and its various useful augmentations, including the original contours that Langan et al.^3^ proposed. It also introduces an illustrative example that will be used throughout the article. Section 3 introduces decision models and explains how they can take advantage of, and be informed by, meta-analyses. Then, Section 4 presents the new methodology developed for augmenting funnel plots based on an additional trial’s ability to change a decision with application to the example introduced in Section 3. Finally, the discussion in Section 5 concludes the article.

## 2 Funnel plots and their applications to date

Meta-analysis is a form of statistical analysis that combines the results of multiple studies to provide an overall summary (usually a weighted average) of the existing evidence for a particular research question. We focus on meta-analysis in a clinical trials context for the remainder of this article. A meta-analysis can be thought of as a quantitative analysis within a systematic review with both fixed- and random-effect models commonly employed, of which details are reported elsewhere.^10^^,^ ^11^

### Illustrative example: Meta-analysis

To demonstrate the methods described in this article, an illustrative example is presented, which is simplified from a previously published analysis.^12^ This example considers RCTs taken from a systematic review^13^ establishing the effectiveness of prophylactic use of neuraminidase inhibitors (NIs) for the reduction of influenza (flu) A and B incidence in healthy adults. The trial results are presented in Table 1. The pooled effect estimate for the relative risk of the NI group contracting flu compared to the control group is 0.28 for both fixed- and random-effects meta-analyses.

**Table 1:** RCTs comparing the incidence of influenza A and B for the prophylactic NI group and the control (placebo) group

	Number in NI group		Number in control	
	contracting	Total number in	group contracting	Total number in
Trial	influenza	NI group	influenza	control group
1	3	268	19	268
2	3	252	6	251
3	11	553	34	554
4	7	414	40	423
5	3	144	9	144

A funnel plot, which was first used to assess publication bias in a meta-analysis, is a scatterplot of multiple trials’ effect estimates and a measure of trial precision (e.g., sample size, standard error, or inverse standard error).^14^ Caution against the uncritical interpretation of funnel plots has been expressed due to alternative possible explanations.^15^ Common features of a funnel plot include the line of no effect, a line indicating the summary effect, and possibly its 95% confidence interval. Pseudo confidence intervals can also be overlayed to show the approximate region in which a certain proportion, usually 95%, of trials would be expected to lie in, assuming that there is no heterogeneity.

A further possible augmentation to the funnel plot is to include contour regions indicating levels of statistical significance of the primary studies displayed on the plot.^16^ This can be helpful for discerning whether any observed asymmetry is due to publication bias, rather than other causes, by considering the statistical significance of the regions of the plot where studies are perceived as missing.

It was suggested by Langan et al.^3^ that, outside of detecting bias and heterogeneity, funnel plots could also be used to illustrate and assess the impact of the addition of a new trial to an existing meta-analysis. They introduced contours that divide the funnel plot into regions based on where a new trial may be located. These include contours for statistical significance, where the regions produced illustrate what combination of effect size and standard error a new trial would need to have to change or maintain the statistical significance of the current meta-analysis.

**Figure 1:**
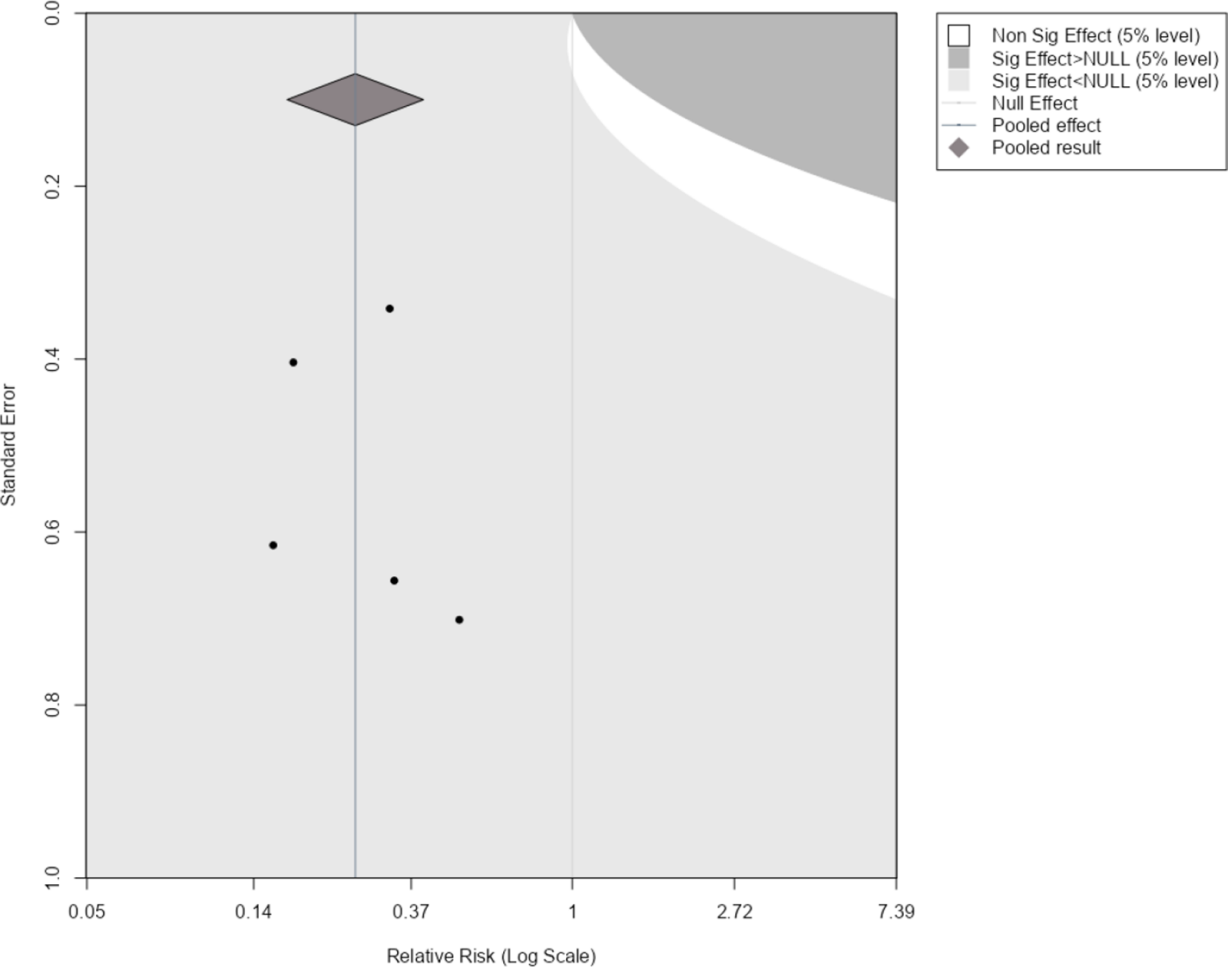
Statistical significance contours (Langan et al.^3^) for the NI-influenza meta-analysis.^13^

Figure 1 shows an example of this applied to our illustrative example. Here, the original meta-analysis is statistically significant in the negative direction at the 5% level as indicated by the pooled effect size 95% confidence interval (diamond) being completely on the left side of the line of no effect. The light grey region indicates where a new trial would have to lie for the updated meta-analysis summary estimate to remain statistically significant in the negative direction. The dark grey region indicates where a new trial would have to lie for the estimate to become statistically significant in the positive direction, and the white region is to become statistically insignificant. By considering where the current studies lie in the plot and the assumption of how similar a new trial may be to the current evidence base, one can get an overall picture of how the meta-analysis may evolve as more evidence accumulates.

## 3 Decision modelling for intervention evaluation

For decision-making in healthcare, clinical effectiveness information alone is often not sufficient for allocating available health care resources to maximise population health. Other factors, such as social, ethical, and economic implications, are considered alongside the medical benefits of a health intervention. While economic evaluations are increasingly conducted alongside RCTs,^17^ findings may be limited by the length of the trial and the intervention options included.^18^ Decision modelling provides an analytical framework that brings together all the relevant evidence required to undertake a comparative analysis of multiple intervention strategies (which may not have been previously trialled together) and enables an assessment of the economic implications, potentially over an extended period of time.

### Illustrative example: Decision model

**Figure 2:**
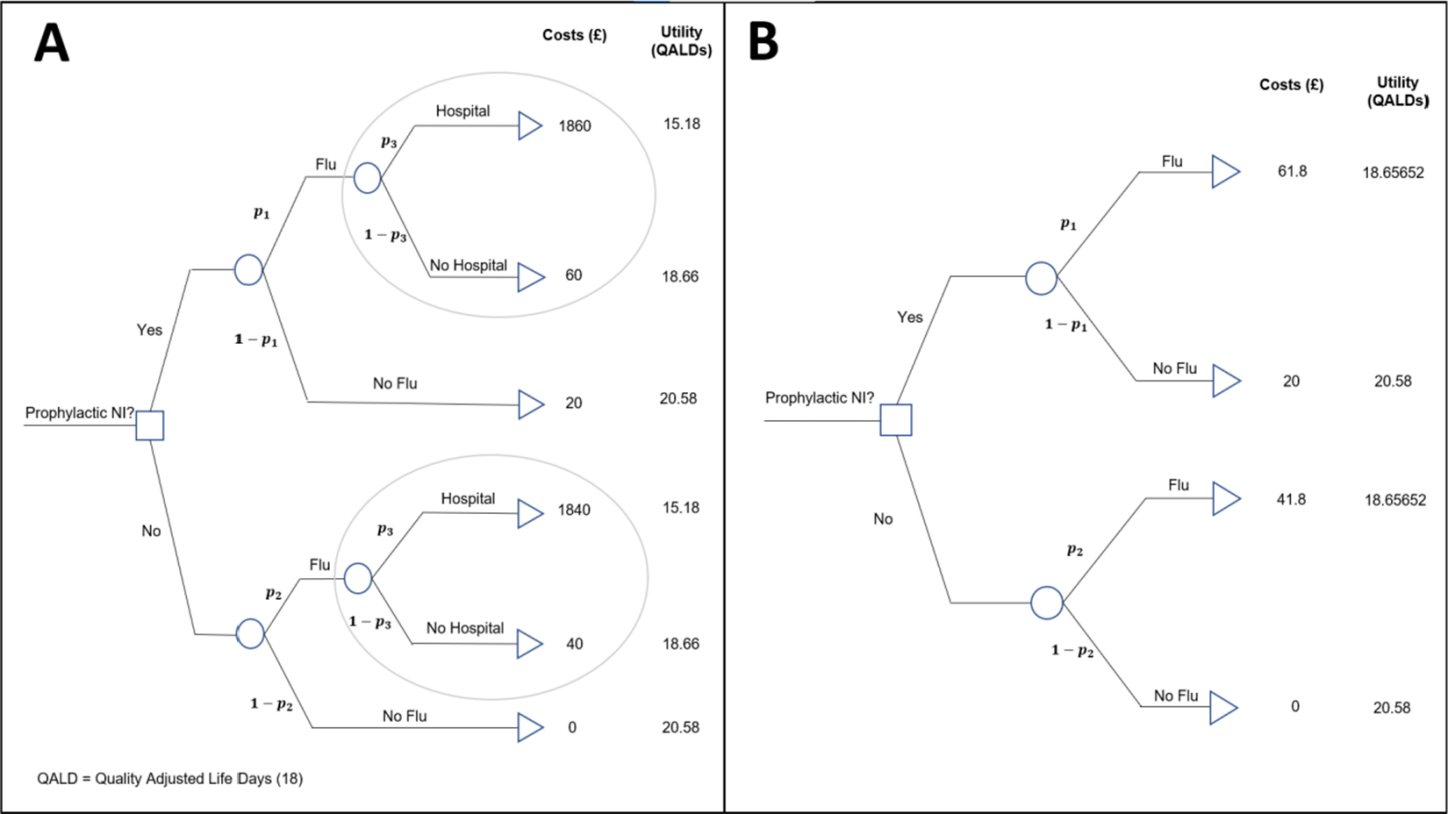
(a) Decision model for evaluating the cost-effectiveness of the prophylactic NI group versus the control group. (b) Reduced decision model for evaluating the cost-effectiveness of the prophylactic NI. Key: 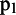 is the probability of a patient contracting the flu given they have been administered NI, 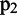 is the baseline probability of a patient contracting flu (i.e., has not been administered NI), and 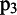 is the probability that a patient is hospitalised given that they are infected with flu. QALDs, quality-adjusted life days.^19^

The example introduced in Section 2 used the meta-analysis conducted by a previous systematic review to inform an economic decision model, to determine if the use of the intervention, prophylactic NI, was cost-effective. The decision tree is presented in Figure 2a.

This is an example of a ‘two-intervention’ decision model, which aims to answer the question: Should a new health intervention be adopted in place of a current, standard intervention? That is, is intervention ‘A’ more cost-effective than intervention ‘B’? In our case, the question is whether prophylactic NI (A) is more cost-effective than the control, no prophylactic NI (B).

The outcome state that patients move to after being allocated an intervention (prophylaxis NI or nothing) is determined by the transition probabilities (e.g., 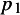 , 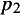 , and 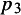 in Figure 2), and each state has a cost and utility associated with it, measured in UK£ and quality-adjusted life days (QALDs). Initially, we ignore uncertainty in the estimation of all decision model parameters, but this is relaxed later in Section 4.3. The costs, utilities, and probabilities assigned to the decision tree are used to calculate expected costs and utilities for each intervention, which are then used to determine which intervention is more cost-effective. The net benefit, *NB*, of an intervention is calculated as
(3.1)

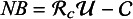



Where 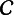 is the expected cost of the intervention across all possible states, 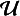 is the expected effects (e.g., QALDs) of the intervention across all states, and 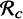 is the willingness to pay (WTP) threshold of the decision-maker. The WTP threshold is the additional cost the decision maker is willing to pay’ to achieve an additional one unit of benefit (utility, i.e., QALD). A higher net benefit means that the intervention is more cost-effective and thus would be chosen over alternative options. In the simple example decision model (Figure 2), the probabilities can be thought of as event probabilities for each intervention group (e.g., probability of contracting a disease given the intervention administration, etc.), and these measures of clinical effectiveness can be informed by pairwise meta-analyses of clinical trials. In the main article, focus will be given to the relative risk scale but equivalent formulae are derived for odds ratios and risk differences in the Appendix of the article. It is noteworthy that, for relative risks and odds ratios, the typical natural logarithm transformation is applied due to better statistical properties.

For the purpose of the methods presented in this article, motivated by the aim of keeping the methodology as general as possible, the decision model presented in Figure 2a has been reduced to a ‘four-branch’ form (two interventions, and one chance node within each intervention that leads to two distinct outcome states) by a partial decision model evaluation ‘rolling back’ the decision tree ‘branches’ highlighted in the ovals in Figure 2a.

This is achieved by calculating expected outcomes for branching points in the tree using the formula for the expected value.
(3.2)

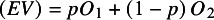



Where *p* is the probability of 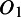 , outcome 1, happening (e.g., flu). And so, 1 − *p* is the probability of 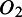 , outcome 2, happening (e.g., no flu). For example, using an estimate of 0.001 for 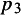 (probability of hospitalisation given the flu, Figure 2a), the expected outcomes of a patient who is:

(i) given NI and contract the flu is:

Expected costs: 0.001 × £1860 + 0.999 × £60 = £61.8.

Expected utilities: 0.001 × 15.18 + 0.999 × 18.66 = 18.65652 QALDs.

(ii) not given NI and contract the flu is:

Expected costs: 0.001 × £1840 + 0.999 × £40 = £41.8.

Expected utilities: 0.001 × 15.18 + 0.999 × 18.66 = 18.65652 QALDs.

This results in the reduced decision tree shown in Figure 2b.

This method of decision tree reduction is a valuable tool that allows more complex decision trees to still utilise the methods developed here for the simple four-branch case. These methods can be applied with relative ease to a generalised class of decision tree structures, also with two interventions and a meta-analysis informing the probability of the outcome occurring ( 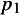 ) in the novel intervention branch. In the example, it is assumed that 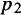 —the probability of the outcome occurring in the no intervention group—is 0.05. This quantity could be derived from an external source, such as routine hospital data in the target population, as is commonly the case for describing ‘usual care’/baseline group in decision modelling.^20^ It is used as opposed to the values of 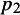 derived from the individual trials as it is considered a better estimate for the decision population and does not break randomisation, as it is unconstrained and makes no assumption about how those probabilities relate to each other. By using a summary estimate of the difference between the two interventions’ clinical effectiveness (derived by the meta-analysis), 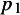 can be derived from any of the binary outcome measures using Equation (3.3). 
(3.3)

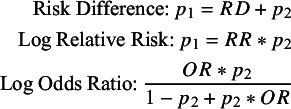



From here, it is now possible to evaluate the net benefits, as defined in Equation (3.1) of each intervention using the decision model. This is achieved by fully ‘rolling back’ the decision tree, calculating the expected costs 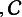 , and effects, 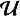 (on the QALD scale) for each intervention group using the same principle as used above for simplifying the model. Then, these estimates are used together with one or more WTP thresholds, *R_C_* (multiple thresholds being useful for exploring robustness of the findings to the decision-maker’s WTP threshold). This will be evaluated for the example dataset within the next section.

## 4 Assessing the impact of future meta-analytic evidence to reverse the existing intervention decision

We will now outline a new methodology developed for augmenting funnel plots based on the ability of an additional trial to change the intervention decision indicated by a decision model, using the example dataset and decision model. When decisions are made using an economic decision model, it is not the statistical significance of an intervention’s effect size that is considered, it is whether the new intervention has a larger net benefit that determines if it is adopted, for example, when interventions are evaluated by NICE in the United Kingdom.^9^ Therefore, under this framework, it is natural to consider such funnel plot augmentations relating to the decision model, instead of the statistical significance of the meta-analysis.

### 4.1 Decision threshold boundaries

#### 4.1.1 Fixed effect

For a decision model informed by a fixed-effects meta-analysis with a binary outcome (log relative risk, log odds ratio, and risk difference), equations for the decision contours relating the combination of a new trial’s effect size and standard error can be explicitly derived. The full derivations can be found in Supplementary Material, Section 1.

**Figure 3:**
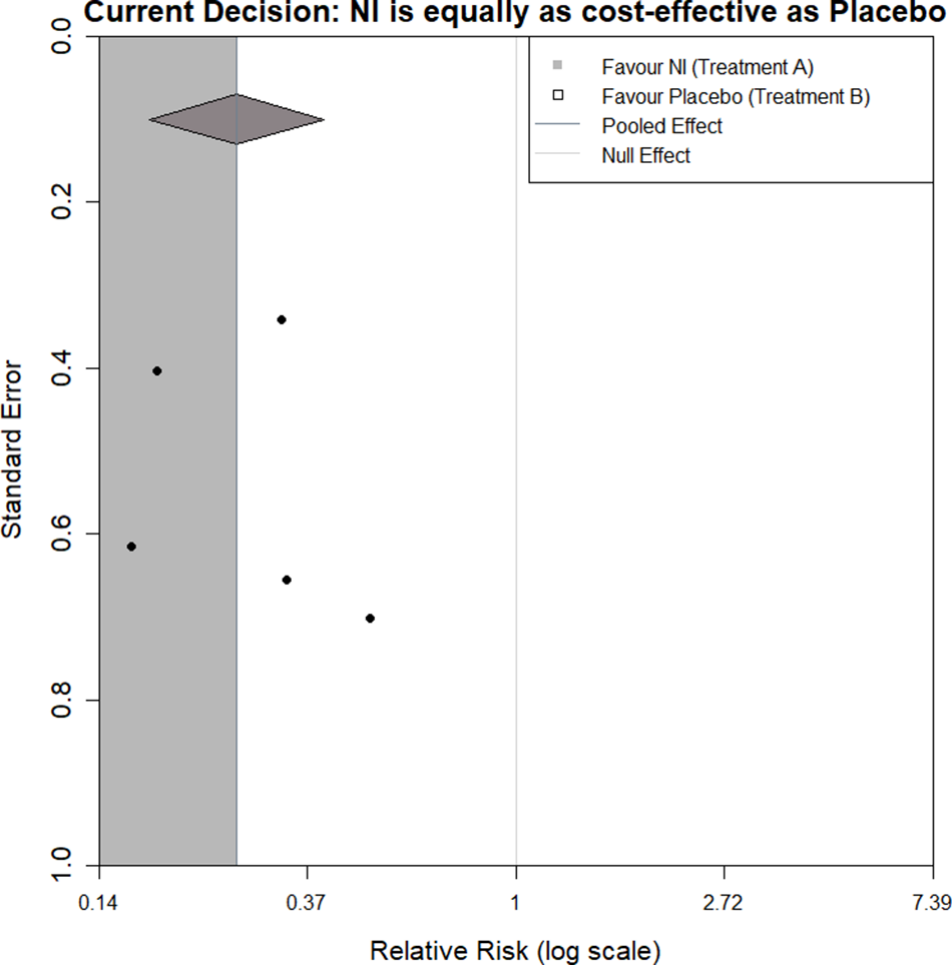
Decision contour for NI versus Control with a log relative risk outcome and WTP value of £260.08 (equal to ICER).

For illustration, we start by considering the case where the current evidence indicates a borderline decision, that is, the two interventions are equal in net benefit. Figure 3 shows a funnel plot of the log relative risks against their standard errors, with the region of impact shaded, as defined by the decision threshold boundary. Figure 3 and all subsequent funnel plots presented in this article were produced using R Statistical Software (v4.1.3; R Core Team 2023).^20^ The code used can be found in the following GitHub repository: https://github.com/WillRobinn/Decision-Contours/tree/main. This is the decision threshold produced given the decision model shown in Figure 2(b), informed by the meta-analysis and used with a WTP value of £260.08 per additional QALD (which is equal to the incremental cost-effectiveness ratio (ICER) for NI vs. control [Supplementary Material, Section 2]). Setting the WTP value to this ICER means that both intervention options are evaluated as equally cost-effective, and an additional trial will change the decision based on which side of the meta-analysis pooled estimate its intervention effect is estimated to be. This means the decision threshold is a vertical line at the pooled estimate.

If a new trial that had a log relative risk and standard error, which lies in the grey shaded region (a trial of any precision that says NI is more effective than the current meta-analysis), was added to the meta-analysis, this would ‘pull’ the net benefit of NI higher than the net benefit of control. However, if the new trial lies in the white region (a trial of any precision that says NI is less effective), this would ‘push’ the net benefit of NI lower than the net benefit of control.

**Figure 4:**
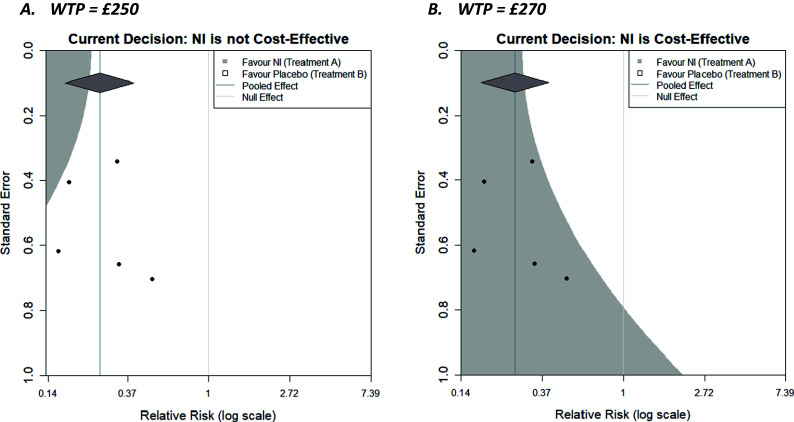
Decision contours for NI versus Control with a log relative risk outcome and WTP values of (a) £250 and (b) £270.

Clearly, the contour displayed will change based on decision model parameters, including the WTP. Recent thinking has proposed that cost-effectiveness thresholds should be based on empirical evidence about health opportunity costs^21^ (compared to being chosen by decision-makers); however, for illustration, we have chosen to display contours for WTPs of £250 and £270 in Figure 4a,b, respectively.

As the WTP value decreases to £250, initial cost-effectiveness analysis suggests that the control is more cost-effective, and given the decision-maker is now willing to pay less for healthcare, NI needs to be more effective to be cost-effective (assuming its cost does not change). This reduces the size of the region in which an additional trial would need to be located to make the decision model favour the NI. Specifically, the region is restricted to the top left corner, where the combination of a relatively precise trial and/or one with an outcome favouring NI (and necessarily increasingly so as the precision of the trial decreases) would be located. As the WTP value increases to £270, initial cost-effectiveness analysis suggests NI is more cost-effective, and since the decision-maker is willing to pay more, the requirements for the result in the new trial become less stringent, and the region in which a new trial could exist and NI still being cost-effective expands. The region in which an additional trial would make the decision model favour the control reduces towards the top right corner, where a trial would decrease the overall effectiveness of NI. Another useful feature of the plots, is that the x-coordinate of the contour corresponding to a standard error of 0 (trial of infinite precision) is the meta-analysis summary outcome that results in a tied-evaluation of intervention and control, that is, the value the meta-analysis needs to result in for intervention and control to be equally cost-effective. Both examples here (i.e., for these particular WTP values) could be described as somewhat robust to the addition of a new trial with respect to the current decision, as all trials lie in the same region in both cases; however, some are close to the boundaries. The plot can also be considered when varying parameters other than the WTP, such as costs and utilities for specific outcomes, or the baseline risk, 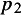 , as part of sensitivity analyses to assess the robustness of the model to changes in parameter values that are uncertain.

#### 4.1.2 Random effects

It is assumed in a random-effects model that the true effect sizes being estimated by each trial are not equal, but belong to a common distribution, with variance τ^2^ (the heterogeneity parameter). The weight of each trial in the meta-analysis is dependent on this variance parameter, which is estimated by the effect sizes and variances of all trials. For this reason, closed-form equations for the decision threshold boundaries cannot be derived as the weights assigned to the trials in the existing meta-analysis will change depending on the intervention estimate and its variance in the new additional trial. Therefore, to create decision contours, it is necessary to utilise numerical methods. The approach taken is to conduct a grid evaluation of the effect size/standard error combination sample space represented in the funnel plot and evaluate the individual locus ‘pixel by pixel.’ While this is computationally relatively straightforward to do, it is more time consuming, computationally expensive, and potentially less insightful. The process is explained in more detail in Supplementary Material, Section 3.

**Figure 5:**
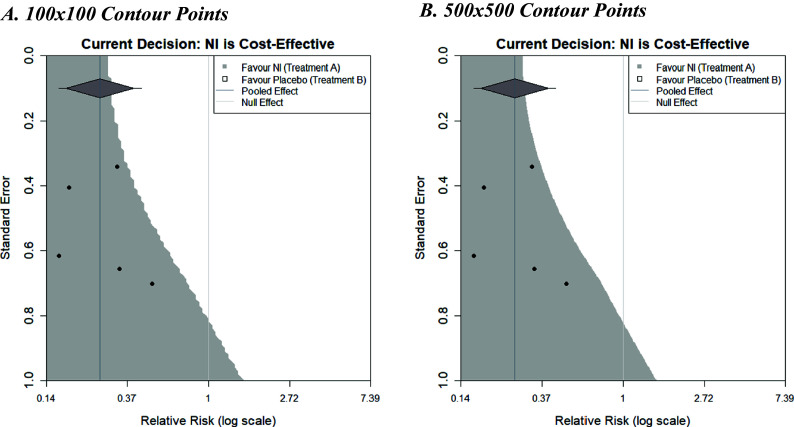
Decision contours for NI versus Control using a random-effects meta-analysis with 100 and 500 contour points.

Figure 5 shows the decision contours generated by the example dataset with a log relative risk outcome, a WTP value of £270, and now informed by a random-effects meta-analysis. The same augmentations are shown as previously, but now that a random-effects meta-analysis is being used, a 95% prediction interval is also displayed as ‘tails’ on the summary diamond.

Plot A used a grid resolution of 100 × 100 points and took 3.3 s to run from the call of the function to the full display of the image on a standard PC. Plot B used a resolution of 500 × 500 points and took 81.2 s to run, but the increased computation time does result in a much smoother boundary threshold, although the additional insight this affords is questionable. It is important to note that the contours generated in Figure 5 differ slightly in shape from the contour shown in Figure 4b. This difference is due to the choice of fixed- or random-effects meta-analysis models (the only difference between the plots). Larger differences could be observed with different examples.

### 4.2 Adding simulated trials of fixed sample size to the plot

**Figure 6:**
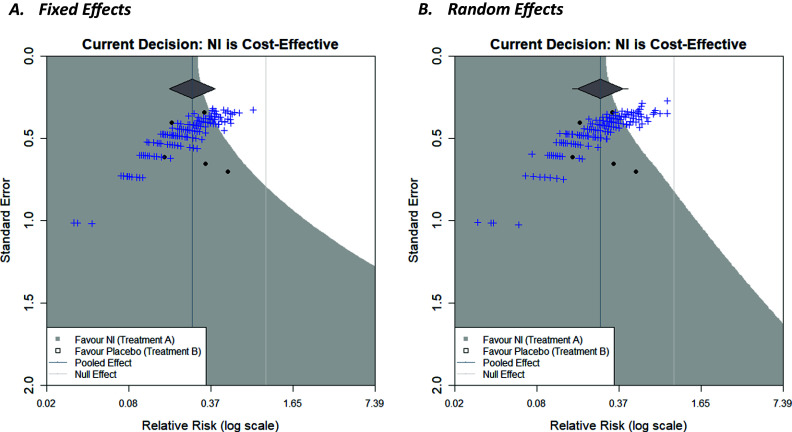
Decision contours for NI versus Placebo with a fixed-effect meta-analysis (a) and random-effects meta-analysis (b) with a log relative risk outcome and WTP value of £270. Overlayed with 200 new simulated trials based on the current meta-analysis, with a sample size of 500 patients per arm.

The decision contour plots, shown thus far, may be useful for a trialist to consider the impact that a new trial may have on a current decision, in addition to the existing evidence base. It would potentially be even more useful to know how the sample size of such a trial would affect this. Sutton et al.^4^ proposed an approach to using an existing meta-analysis to inform sample size calculations of a future trial that subsequently will be included in an updated version of the meta-analysis. Essentially, a distribution for the effect size of a new trial, predicted from the existing meta-analysis, can be used to simulate a new trial. An effect size is realised from this distribution. Then, for a given sample size, data for the new trial is generated stochastically using this effect size, which allows for variation in the potential outcome of the new trial due to sampling error. The trial is then included in the meta-analysis, which is then re-meta-analysed, and whether the conclusions of the meta-analysis change or not is recorded. Repeating this whole simulation procedure for many replications can give an estimate of the ‘power’ of a new trial to change conclusions by calculating the proportion of simulated trials for which a particular conclusion is observed. More details can be found in Sutton et al.^4^ Langan et al.^3^ used this approach to overlay the many simulations of future trials, for a given sample size, onto the funnel plots, giving a visual representation, and thus further insight, of this “power” by showing how many of the trials are in each shaded region and how close they are to region boundaries, and so forth. The same approach can be taken with the decision contours, as shown in Figure 6.

Decision contours for both a fixed-effect meta-analysis (Figure 6a) and a random-effects meta-analysis (Figure 6b) with a log relative risk outcome and WTP value of £270 have been shown. In each case, the current decision is that NI is cost-effective. Two-hundred trials have been simulated, with a sample size of 500 patients per arm, using the results of the respective meta-analyses, and are represented by the blue crosses. As only a small proportion of these lie in the white region in both cases, this signifies that it is unlikely for a new trial of this magnitude to change the current decision reflecting low ‘power’, but the chance is non-negligible (this chance could be quantified by counting the trials in the white region). A natural next step would be to repeat this for different sample sizes which would allow researchers to compare the ‘power’ to change a decision for future trials with different sample sizes (a similar exercise, but regarding the power to change statistical significance of the meta-analysis, has been conducted by creating an interactive app, MetaImpact,^22^ to trial and compare different sample sizes). The number of replications conducted can be increased to increase the precision of the estimate of ‘power’ as required, and so forth. Additional contours, similar to that in Figure 5 in Langan et al.,^3^ could be overlayed to show the range of effect size/standard error combinations of differing sample sizes within new trials (assuming a fixed event rate in the control group). The correlation between the effect size and precision is evident in the curvature of the plot cloud in Figure 3 above.

### 4.3 Incorporating parameter uncertainty

The simplest decision that models take is a deterministic approach, which treats all parameters as known, with no uncertainty, and this is the type presented above. However, incorporating uncertainty in a decision model can be very important as this reflects the uncertainty in the remaining model parameters (i.e., in addition to effectiveness) and thus the current uncertainty about cost-effectiveness. This can change the overall conclusion^23^ when the specified probability distribution for a parameter is non-symmetric, or when the model uses non-linear functions of input parameters.^24^ It also allows the quantification of the probability that a decision is correct and helps decide if further collection of information is worthwhile. Uncertainty can be incorporated for the various parameters in a decision model, such as the costs, utilities, baseline probability, and meta-analysis outcome by specifying statistical distributions, representing current knowledge about the parameters. Once the distributions are fit to the model parameters, the decision model is evaluated many times, sampling different parameter values from the distributions at each iteration; this is called a Monte Carlo simulation. The decision model is then evaluated as before, but now at each iteration, and the combination of all of these results allows uncertainty in the decision to be calculated. For example, if 90% of the sample iterations concluded that the new intervention was cost-effective, then the probability of that new intervention being cost-effective would be 0.9.

**Figure 7:**
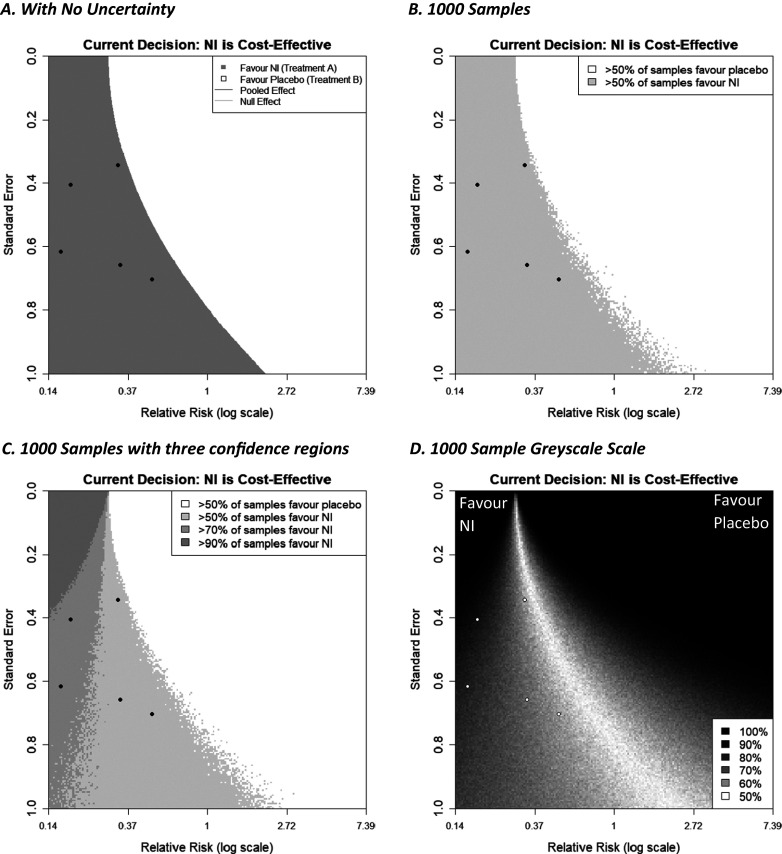
Decision contours (a) no uncertainty in the decision model, (b, c, d) 1000 samples of a Monte Carlo simulation to incorporate uncertainty of the meta-analysis effect estimate into the decision model. 7(b) categorises new studies by two regions, (c) by 4 regions, (d) using a greyscale of many shades.

Below, we illustrate how decision model parameter uncertainty can be considered and presented on a funnel plot when considering the impact of a new study on the decision for prophylaxis NI to prevent influenza. For simplicity, in this example, we incorporate the uncertainty in the pooled estimate from the meta-analysis only, but uncertainty in all model parameters, such as 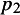 , could also be incorporated using the same process. Such calculations increase computation time considerably because now the evaluation of each locus in the funnel plot grid sample space needs to use many iteration samples drawn from the parameter distribution(s) as described above. In this way, the probability that NIs are cost-effective is calculated for each locus. If a particular threshold is defined, for example, >50% of the evaluated samples indicate NIs are cost-effective, then each locus can be coloured in when this condition is satisfied (or left white otherwise). This is done in Figure 7b using a fixed-effect meta-analysis with a log relative risk outcome, a WTP value of 270, and a 200 × 200 grid of points. One hundred samples are drawn from the meta-analysis parameter estimate (in this case, the log relative risk) distribution (which is assumed to be normally distributed), and for each one, the decision model is evaluated. Loci are coloured grey if more than 50% of these stochastic samples favoured NI to control, and white otherwise. Figure 7b shows this alongside the equivalent plot with no uncertainty (Figure 7a).

The general shape of the shaded regions in Figure 7b is similar to the plot with no uncertainty added (Figure 7a), although now there is some fuzzy ‘noise’ on the boundary threshold, particularly where the additional trial has a large standard error. As more samples are drawn, this noise decreases and would eventually disappear, leaving a smooth boundary if more and more samples were utilised. While the resulting curve would appear to be similar to that generated ignoring uncertainty, this will be example-specific and not always the case. It is emphasised that uncertainty can be applied to other decision model parameters, such as the baseline probability 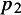 . The current example code currently only supports uncertainty for the meta-analysis estimate, but with sufficient programming knowledge, other parameters can be considered.

Extending this concept further, we can create multiple shaded regions that correspond to different thresholds of confidence, for example, a region where more than 50% of samples favour NI, a region where more than 70% of samples favour treatment NI, and 90% of samples, and so forth. These can also be thought of as regions corresponding to a particular probability of cost-effectiveness of the intervention NI. This is shown in Figure 7c. This could also be done for thresholds lower than 50%, which would give us more detailed information on the unshaded regions of the plot, consequently telling us about the regions in which the cost-effectiveness of the current treatment changes.

To have as much information as possible about the probability of cost-effectiveness, in Figure 7d, each locus on the plot is assigned a value measured on a scale from 1 to 100, based on the percentage of simulations that favoured the NI intervention. To illustrate the uncertainty in *either* direction, a diverging colour scheme was utilised—we chose greyscale for its simplicity and colour-blind friendliness, but other diverging colour schemes can be used, including ones where the colour is different for each intervention. As we chose the colour to be the same (i.e., black) for both interventions, values for loci that were initially assigned a value of <50 had to be ‘reversed’ (i.e., new value = 100 – old value). Then, by assigning colours to the loci based on their value (here 100 = black and 50 = white), we create a ‘heat map’-like plot, with black representing intervention decision certainty for either intervention (NI or no intervention in this case) and lighter shades increasingly representing decision uncertainty.

In this plot, the darker the shade, the more samples favoured one of the interventions, with the legend labelling that percentage of samples. This plot provides further insight into how certain a decision would be when considering new studies with particular effect size/standard error combinations. Here, three of the five existing studies are located in a region, indicating considerable uncertainty, suggesting that one further similar study may not reduce decision uncertainty very much. As discussed in Section 4.1.2, it should be noted that changing the meta-analysis model from fixed effects to random effects can greatly change what shade of grey each locus is shaded.

It is noteworthy that if parameters are represented by distributions as explained here, and the decision model has been reduced (i.e., similar to that shown in Figure 2) with such parameters in the ‘reduced’ section of the tree, then the resulting decision tree will be an approximation to the original decision model (see Section 5 for further consideration).

## 5 Discussion

In this article, we have described in detail how the approach to generate statistical significance contours on a funnel plot to illustrate the potential impact of a new study on a meta-analysis (as proposed by Langan et al.^3^) can be extended to a decision-making context. In the decision contour plot proposed here, the regions and contours are constructed to show the impact that an intervention effect estimate from a new study will have when the updated meta-analysis is used to inform an economic decision model. More specifically, the decision contour plot relates to a ‘four-branch’ decision model that concerns two interventions (usually a new intervention being compared against a standard intervention or usual care), each with one clinical event that leads to two distinct outcome states (death or no death, ill or not ill, etc.), where clinical effectiveness of the two interventions is informed by a fixed-effect or random-effects meta-analysis on the log relative risk, log odds ratio, or risk difference scale.

The methods, as presented here, do have some limitations, including those already described for the statistical significance contours in Langan et al.^3^: (i) The plots are only capable of showing the impact of a single additional trial, whereas the impact of multiple further trials may be of interest; (ii) the impact of two trials could be assessed using a three-dimensional (3D) plot; however, any number of trials beyond this is unlikely to be easily visualisable, and the interpretation of the 3D plot alone may be difficult; and (iii) the plots do not consider a change in meta-analysis model, which could occur if the level of heterogeneity is increased from new trials (i.e., moving from fixed to random effects). Further limitations specific to the decision contour plot proposed here include the focus on binary outcomes in this article, although we believe that both the analytical and numerical methods described could be generalised quite easily for continuous outcomes. Further, the plots using fixed-effect meta-analysis are based on inverse-variance weighted methods, but, again, the methods such as Mantel–Haenszel and Peto methods for specific binary meta-analysis outcome measures could be used.^3^^,^ ^11^

Although the methods are generalisable to a class of two-intervention decision trees, there exist more complex decision models that answer more complex health research questions. For example, there may be more than two interventions of interest. An extension of the plot to allow for this could perhaps be having multiple shaded regions, each one representing outcome/precision combinations for the new trial that would make the decision model overall favour one of those interventions. Often, network meta-analysis,^25^ which allows for simultaneous comparison of multiple interventions, will inform such decision models. It should be noted that, even without the use of a decision model, there is work to be done representing the impact of a further trial on a network meta-analysis. Network meta-analysis can not only establish which intervention is the most beneficial, but also rank them in a hierarchy and provide the probability that this ranking assignment is correct. This information could result in various augmentations on a funnel plot, such as a colour to represent the intervention that the inclusion of a new trial determines is best, and the opacity of such a colour representing the probability that this evaluation is correct. Alternatively, multiple plots could be presented for each intervention, with a colour representing the most likely ranking for that intervention when the new trial is added to the analysis. Furthermore, integration of network meta-analysis into the current methodology would be beneficial even for the two-intervention case, as it allows for the inclusion of indirect evidence between the two interventions, which could improve the accuracy of the contours. Again, this could also be implemented for the statistical significance contours.

While application of the plot to the two-intervention ‘four-branch’ class of decision trees may seem to restrict the current methodology’s application to a small set of decision models, the method of decision tree reduction illustrated in Section 4.1 allows these methods to be applied to more complex decision trees that compare two interventions. As long as it is possible to ‘roll back the tree’ to the form shown in Figure 2 by averaging the effect of the decisions further along the tree, the same results can be obtained if the tree is deterministic beyond the reduction point (set values for each parameter), or approximated if the tree is stochastic beyond the reduction point (i.e., parameters informed by distributions). However, not all decision models can be reduced to this form. For example, another commonly used class of more complex decision models is Markov models.^26^ Technically, Markov models can be represented as decision trees, so the reduction method described here could possibly be implemented. However, they are often very large and contain many events. Depending on the model, the calculations to reduce such a tree may be burdensome. However, this and many of the previously identified shortcomings are not a limitation of the methodological approach per se, just our initial implementation of it. It would be possible to extend the simulation-based approach this work utilises to any specific decision model and it could be informed by multiple evidence syntheses using different models (e.g., network meta-analysis, etc.) using different outcome scales from those considered here (e.g., standardised mean difference) but it would take bespoke computer code to do it. For complex decision models, the computational time required to generate the plots may be substantial; however, since the evaluation of each locus on a plot is both independent and uses the same equations, such a method would be ripe for a parallel processing implementation either using multi-core CPUs or via Graphics Processing Units.

Statistical concepts can be difficult to communicate to non-specialists, especially when presented with a large number of figures.^27^ A method of tackling this is through accessible interactive graphical displays, where a user can vary parameters within a plot and see how it changes in real time. It has already been highlighted that generating the decision contours for various parameter values would be useful for purposes such as sensitivity analyses. Developing interactive versions of the plots, by creating sliders for decision model parameters, for example, would help make the interpretation of these plots easier for statisticians, as well as non-specialists. Therefore, it would be useful for decision-makers to be able to explore how the contours would change with respect to different assumptions about model parameter values (and their level of uncertainty), including those which can suddenly change, such as an intervention cost, or WTP. Decision-makers may also use these plots to prioritise updating of technology appraisals in the advent of new evidence becoming available, in a similar vein to the updating of portfolios of meta-analyses.^5^ In Section 1, traditional hypothesis test-driven approaches to sample size calculations and EVSI approaches were briefly outlined, and the similarities and differences with the approach presented herein highlighted. Despite their dominance in practice, traditional sample size calculations take a very narrow perspective, considering only the statistical significance of the primary outcome of the future trial. At the other end of the spectrum are EVSI calculations, which take a much broader perspective and ultimately answer the question of whether the future trial would be good value for money while taking into consideration the vast array of relevant clinical and economic factors. These latter calculations, while being comprehensive, are not without their drawbacks. They are invariably lengthy, difficult to understand intuitively, and assume knowledge of often difficult to estimate quantities, such as the effective lifetime of an intervention (i.e., before it is superseded).^8^ There is clearly a huge gulf between these two approaches and the plots presented here (as were those by Langan et al.^3^) provide intermediary perspectives, and, due to their graphical nature, provide insight into the underlying calculations.

When designing a new trial, there may be a role for the simultaneous consideration of multiple approaches. For example, both the statistical significance contours developed by Langan et al.,^3^ the decision-based contours, and EVSI calculations may be of interest when developing a new drug. Pharmaceutical companies are concerned with both obtaining licensing approval and recommendation for use in health-care systems through evaluations like those required by NICE in England and Wales. The former considers criteria based around statistical significance, while cost-effectiveness is also required for the latter, making the decision contours relevant. Hence, it may be valuable to ascertain the impact a new study is likely to have on both criteria before finalising a design, and EVSI could be utilised to ascertain whether paying for such a trial would be good value for money for the company. Thus, we are not suggesting one approach is always superior to another, but believe further experience in the simultaneous consideration of multiple approaches is an interesting research avenue.

## Supporting information

Robinson et al. supplementary material Robinson et al. supplementary material

## Data Availability

No individual-level data were used in the work conducted in this article.

## Supplementary material

To view supplementary material for this article, please visit http://doi.org/10.1017/rsm.2025.10011.
