# Supplementary material for: Exploring graphical approaches to assess the impact of an additional trial on a decision model via updated meta-analysis
Source: Res Synth Methods. 2025 Jun 4;16(4):672–87. doi: 10.1017/rsm.2025.10011 (PMC12527514; doi:10.1017/rsm.2025.10011)
Supplement: Robinson et al. supplementary material [file S1759287925100112sup001.docx]

**Supplementary Material**

1. Calculation of ICER and subsequent WTP value:

The following derivations are based on the general 4-branch decision tree as shown in Figure S1.1


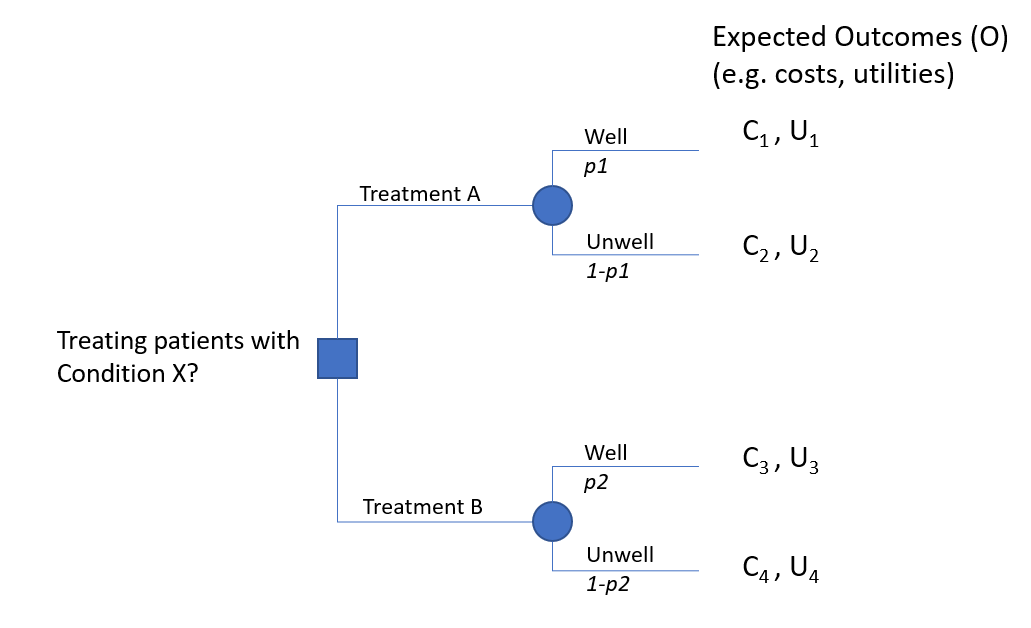


Figure S1.1: General 4-branch decision tree

The willingness to pay (WTP) threshold of the decision maker is a key parameter and will have a large impact on what decision is made, for the current meta-analysis, as well as many potential additional trials. An equivalent method of determining cost-effectiveness to the net benefit of interventions, is to use the incremental cost-effectiveness ratio (ICER), which is calculated as:

| (4.6) | $\frac{C_{A}-C_{B}}{U_{A}-U_{B}}=\frac{\left( p_{1}C_{1}+\left( 1-p_{1} \right)C_{2} \right)-(p_{2}C_{3}+\left( 1-p_{2} \right)C_{4})}{\left( p_{1}U_{1}+\left( 1-p_{1} \right)U_{2} \right)-(p_{2}U_{3}+\left( 1-p_{2} \right)U_{4})}$ | (S1.1) |
| --- | --- | --- |

If the ICER is below the WTP value, then the new intervention (A) is deemed as cost-effective compared to the control (B), and it is too expensive if the ICER is above the WTP. If we use the current meta-analysis to calculate $p_{1}$, then we can calculate the current ICER. Setting our WTP value to this ICER means that both intervention options will be as cost-effective as each other, and an additional trial will change the decision based on where it is in relation to the pooled estimate. This means the decision contour will be a vertical line at the pooled estimate. The existing meta-analysis gives a pooled log relative risk of −1.339096, using equation (4.7), we can calculate the ICER:

$$\frac{\left( p_{1}C_{1}+\left( 1-p_{1} \right)C_{2} \right)-\left( p_{2}C_{3}+\left( 1-p_{2} \right)C_{4} \right)}{\left( p_{1}U_{1}+\left( 1-p_{1} \right)U_{2} \right)-\left( p_{2}U_{3}+\left( 1-p_{2} \right)U_{4} \right)}$$

$$=\frac{\left( {RRp}_{2}C_{1}+\left( 1-{RRp}_{2} \right)C_{2} \right)-\left( p_{2}C_{3}+\left( 1-p_{2} \right)C_{4} \right)}{\left( RRp_{2}U_{1}+\left( 1-{RRp}_{2} \right)U_{2} \right)-\left( p_{2}U_{3}+\left( 1-p_{2} \right)U_{4} \right)}$$

$$=\frac{(exp \left( -1.339096 \right)*0.05*61.8+\left( 1-\exp\left( -1.339096 \right)*0.05 \right)*20)-(0.05*41.8+\left( 1-0.05 \right)*0)}{(exp \left( -1.339096 \right)*0.05*18.65652+\left( 1-\exp\left( -1.339096 \right)*0.05 \right)*20.58)-(0.05*18.65652+\left( 1-0.05 \right)*20.58)}$$

$$=£260.0838$$

1. Derivation of fixed effects contours

We seek a contour that shows the boundary of where a new trial would have to be to change the decision of a cost-effectiveness model, i.e., where the net benefit of two treatments are equal or “tied-evaluation”. The difference in treatment clinical effectiveness ($p_{1}vs p_{2}$) is informed by an existing meta-analysis of $n$ trials. The bound of the contour is given by:

| (2.1) | $\hat{\theta}= \theta_{0}$ | (S2.1) |
| --- | --- | --- |

Where $\hat{\theta}$ is the summary estimate of the meta-analysis including the new trial given by:

| (2.1) | $\hat{\theta}=\frac{\sum_{i=1}^{n} \omega_{i}\hat{\theta}_{i}+\omega_{new}\hat{\theta}_{new}}{\sum_{i=1}^{n} \omega_{i}+\omega_{new}}$ | (S2.2) |
| --- | --- | --- |

$\hat{\theta}_{i}=$ effect size of trial $i$

$\omega_{i}=$weight of trial $i$

$\hat{\theta}_{new}=$ effect size of new trial

$\omega_{new}=$weight of new trial

And where $\theta_{0}$ is the value of tied-evaluation i.e., the value that the summary outcome must be for the net benefit of both treatments to be the same, this depends on what summary measure is being used, as well as the costs and utilities, the willingness to pay threshold, and the probability of an event for treatment B (current treatment or “standard care”).

Substituting (S2.2) in to (S2.1) and rearranging:

$$\frac{\sum_{i=1}^{n} \omega_{i}\hat{\theta}_{i}+\omega_{new}\hat{\theta}_{new}}{\sum_{i=1}^{n} \omega_{i}+\omega_{new}}=\theta_{0}$$

$$\sum_{i=1}^{n} \omega_{i}\hat{\theta}_{i}+\omega_{new}\hat{\theta}_{new}=\theta_{0}\left( \sum_{i=1}^{n} \omega_{i}+\omega_{new} \right)$$

$$\omega_{new}\hat{\theta}_{new}=\theta_{0}\left( \sum_{i=1}^{n} \omega_{i}+\omega_{new} \right)-\sum_{i=1}^{n} \omega_{i}\hat{\theta}_{i}$$

|  | $\hat{\theta}_{new}=\frac{1}{\omega_{new}}\left( \theta_{0}\left[ \sum_{i=1}^{n} \omega_{i}+\omega_{new} \right]-\sum_{i=1}^{n} \omega_{i}\hat{\theta}_{i} \right)$ | (S2.3) |
| --- | --- | --- |

Here we have derived the effect size that a new trial with a given weight (which is based on the trial precision) should have to be on the boundary between regions where each treatment is favoured by the decision model, i.e., for the net benefit of the treatments to be equal (tied-evaluation). Now we will show how to write this in terms of the decision model.

The net benefit of Treatment A, $NB_{A}$, and Treatment B, $NB_{B}$, are defined as:

${NB}_{A}=R_{c}U_{A}-C_{A}$

$$NB_{B}=R_{c}U_{B}-C_{B}$$

Where:

$U_{A}$= Expected utilities upon receiving treatment A

$C_{A}$= Expected costs upon receiving treatment A

$U_{B}$= Expected utilities upon receiving treatment B

$C_{A}$= Expected costs upon receiving treatment B

$R_{c}$ = Willingness to pay for an additional unit of benefit (in our case, £s per QALD)

For a tied-evaluation the net benefit of each treatment needs to be the same:

| (4.4) | ${NB}_{A}=R_{c}U_{A}-C_{A}= NB_{B}=R_{c}U_{B}-C_{B}$ | (S2.4) |
| --- | --- | --- |

We can calculate $C_{A}, C_{B}, U_{A}$ and $U_{B}$ by “rolling back the tree” to the first decision in the decision tree, since the outcomes for each state are known:

| (4.5) (4.5) (4.5) (4.5) | $U_{A}=p_{1}U_{1}+\left( 1-p_{1} \right)U_{2}$  $C_{A}=p_{1}C_{1}+\left( 1-p_{1} \right)C_{2}$  $U_{B}=p_{2}U_{3}+\left( 1-p_{2} \right)U_{4}$  $C_{B}=p_{2}C_{3}+\left( 1-p_{2} \right)C_{4}$ | (S2.5) (4.5) (4.5) (4.5) |
| --- | --- | --- |

From which the quantities $p_{1}$, $p_{2}$, $C_{1}$, $C_{2}$, $U_{1}$ and $U_{2}$ are based on Figure S2.1.

Substituting these into equation (S2.4):

$$R_{c}\left( p_{1}U_{1}+\left( 1-p_{1} \right)U_{2} \right)-\left( p_{1}C_{1}+\left( 1-p_{1} \right)C_{2} \right)=R_{c}\left( p_{2}U_{3}+\left( 1-p_{2} \right)U_{4} \right)-\left( p_{2}C_{3}+\left( 1-p_{2} \right)C_{4} \right)$$

And in an attempt to simplify this expression, we factor out $p_{1}$ and $p_{2}$:

$$p_{1}\left( R_{c}U_{1}-C_{1} \right)+\left( 1-p_{1} \right)\left( R_{c}U_{2}-C_{2} \right)=p_{2}\left( R_{c}U_{3}-C_{3} \right)+\left( 1-p_{2} \right)\left( R_{c} U_{4}-C_{4} \right)$$

$$p_{1}\left( R_{c}U_{1}-C_{1} \right)+R_{c}U_{2}-C_{2}-p_{1}\left( R_{c}U_{2}-C_{2} \right)=p_{2}\left( R_{c}U_{3}-C_{3} \right)+R_{c} U_{4}-C_{4}-p_{2}\left( R_{c} U_{4}-C_{4} \right)$$

$$p_{1}\left( R_{c}U_{1}-C_{1}-R_{c}U_{2}+C_{2} \right)+R_{c}U_{2}-C_{2}=p_{2}\left( R_{c}U_{3}-C_{3}-R_{c}U_{4}+C_{4} \right)+R_{c}U_{4}-C_{4}$$

$$p_{1}\left( R_{c}\left( U_{1}-U_{2} \right)-\left( C_{1}-C_{2} \right) \right)+R_{c}U_{2}-C_{2}=p_{2}\left( R_{c}\left( U_{3}-U_{4} \right)-\left( C_{3}-C_{4} \right) \right)+R_{c}U_{4}-C_{4}$$

This equation does not reduce easily. For now, we will isolate $p_{1}$:

$$p_{1}\left( R_{c}\left( U_{1}-U_{2} \right)-\left( C_{1}-C_{2} \right) \right)=p_{2}\left( R_{c}\left( U_{3}-U_{4} \right)-\left( C_{3}-C_{4} \right) \right)+R_{c}\left( U_{4}-U_{2} \right)-\left( C_{4}-C_{2} \right)$$

| (4.6) | $p_{1}= \frac{p_{2}\left( R_{c}\left( U_{3}-U_{4} \right)-\left( C_{3}-C_{4} \right) \right)+R_{c}\left( U_{4}-U_{2} \right)-\left( C_{4}-C_{2} \right)}{R_{c}\left( U_{1}-U_{2} \right)-\left( C_{1}-C_{2} \right)}$ | (S2.6) |
| --- | --- | --- |

This is the full relationship between $p_{1}$, $p_{2}$ and the rest of the parameters in the decision model that holds during a tied-evaluation. The way this relates to the meta-analysis summary estimate is dependent on what outcome measure is being used. I will now explore these outcome measures in greater detail.

Risk Difference

Risk difference $RD$, is defined as the difference in probability of an event for two treatments, in our case the risk difference for Treatment A is:

$$RD=p_{1}-p_{2}$$

Substituting for $p_{1}$ using equation (S2.6) I come up with a new equation for the tied-evaluation:

$$\theta_{0}=RD=\frac{p_{2}\left( R_{c}\left( U_{3}-U_{4} \right)-\left( C_{3}-C_{4} \right) \right)+R_{c}\left( U_{4}-U_{2} \right)-\left( C_{4}-C_{2} \right)}{R_{c}\left( U_{1}-U_{2} \right)-\left( C_{1}-C_{2} \right)}-p_{2}$$

Substituting this into equation (S2.3) I get the full definition of the decision contour for the addition of a new trial under the risk difference:

$\theta_{new}=\frac{1}{\omega_{new}}\left[ (\sum_{i=1}^{n} \omega_{i}+\omega_{new})\left( \frac{p_{2}\left( R_{c}\left( U_{3}-U_{4} \right)-\left( C_{3}-C_{4} \right) \right)+R_{c}\left( U_{4}-U_{2} \right)-\left( C_{4}-C_{2} \right)}{R_{c}\left( U_{1}-U_{2} \right)-\left( C_{1}-C_{2} \right)}-p_{2} \right)-\sum_{i=1}^{n} \omega_{i}\hat{\theta}_{i} \right]$

Log Relative Risk

Relative risk $RR$ is defined as the ratio of probability of an event for two treatments, in our case the relative risk for treatment A is:

$$RR = \frac{p_{1}}{p_{2}}$$

Substituting equation (S2.6) into this I can come up with a new equation for the tied-evaluation:

$$\theta_{0}=RR=\frac{\frac{p_{2}\left( R_{c}\left( U_{3}-U_{4} \right)-\left( C_{3}-C_{4} \right) \right)+R_{c}\left( U_{4}-U_{2} \right)-\left( C_{4}-C_{2} \right)}{\left( R_{c}\left( U_{1}-U_{2} \right)-\left( C_{1}-C_{2} \right) \right)}}{p_{2}}$$

$$= \frac{p_{2}\left( R_{c}\left( U_{3}-U_{4} \right)-\left( C_{3}-C_{4} \right) \right)+R_{c}\left( U_{4}-U_{2} \right)-\left( C_{4}-C_{2} \right)}{p_{2}\left( R_{c}\left( U_{1}-U_{2} \right)-\left( C_{1}-C_{2} \right) \right)}$$

This can be transformed for the log relative risk under the condition that the above quantity is positive:

$$\theta_{0}=ln(RR)=ln\left( \frac{p_{2}\left( R_{c}\left( U_{3}-U_{4} \right)-\left( C_{3}-C_{4} \right) \right)+R_{c}\left( U_{4}-U_{2} \right)-\left( C_{4}-C_{2} \right)}{p_{2}\left( R_{c}\left( U_{1}-U_{2} \right)-\left( C_{1}-C_{2} \right) \right)} \right)$$

Substituting this into equation (S2.3) I get the full definition of the decision contour for the addition of a new trial under the log relative risk:

$\theta_{new}=\frac{1}{\omega_{new}}\left[ \left( \sum_{i=1}^{n} \omega_{i}+\omega_{new} \right)ln\left( \frac{p_{2}\left( R_{c}\left( U_{3}-U_{4} \right)-\left( C_{3}-C_{4} \right) \right)+R_{c}\left( U_{4}-U_{2} \right)-\left( C_{4}-C_{2} \right)}{p_{2}\left( R_{c}\left( U_{1}-U_{2} \right)-\left( C_{1}-C_{2} \right) \right)} \right)-\sum_{i=1}^{n} \omega_{i}\hat{\theta}_{i} \right]$

Log Odds Ratio

Odds Ratio is defined as the ratio of odds of an event for two treatments, in our case the odds ratio for treatment A is:

$$OR=\frac{odds(Event in Treatment A)}{odds(Event in Treatment B)}=\frac{odds(A)}{odds(B)}=\frac{\frac{p_{1}}{1-p_{1}}}{\frac{p_{2}}{1-p_{2}}}=\left( \frac{1-p_{2}}{p_{2}} \right)\left( \frac{p_{1}}{1-p_{1}} \right)$$

Substituting equation (S2.6) into this I can come up with a new equation for the tied-evaluation:

$$\theta_{0}=OR=\left( \frac{1-p_{2}}{p_{2}} \right)\left( \frac{\frac{p_{2}\left( R_{c}\left( U_{3}-U_{4} \right)-\left( C_{3}-C_{4} \right) \right)+R_{c}\left( U_{4}-U_{2} \right)-\left( C_{4}-C_{2} \right)}{p_{2}\left( R_{c}\left( U_{1}-U_{2} \right)-\left( C_{1}-C_{2} \right) \right)}}{1-\frac{p_{2}\left( R_{c}\left( U_{3}-U_{4} \right)-\left( C_{3}-C_{4} \right) \right)+R_{c}\left( U_{4}-U_{2} \right)-\left( C_{4}-C_{2} \right)}{p_{2}\left( R_{c}\left( U_{1}-U_{2} \right)-\left( C_{1}-C_{2} \right) \right)}} \right)$$

This can be transformed for the log odds ratio under the condition that the above quantity is positive:

$$\theta_{0}=ln(OR)=ln\left( \left( \frac{1-p_{2}}{p_{2}} \right)\left( \frac{\frac{p_{2}\left( R_{c}\left( U_{3}-U_{4} \right)-\left( C_{3}-C_{4} \right) \right)+R_{c}\left( U_{4}-U_{2} \right)-\left( C_{4}-C_{2} \right)}{p_{2}\left( R_{c}\left( U_{1}-U_{2} \right)-\left( C_{1}-C_{2} \right) \right)}}{1-\frac{p_{2}\left( R_{c}\left( U_{3}-U_{4} \right)-\left( C_{3}-C_{4} \right) \right)+R_{c}\left( U_{4}-U_{2} \right)-\left( C_{4}-C_{2} \right)}{p_{2}\left( R_{c}\left( U_{1}-U_{2} \right)-\left( C_{1}-C_{2} \right) \right)}} \right) \right)$$

Substituting this into equation (S2.3) I get the full definition of the decision contour for addition of a new trial under the log odds ratio:

$$\theta_{new}=\frac{1}{\omega_{new}}\left[ \left( \sum_{i=1}^{n} \omega_{i}+\omega_{new} \right)\ln\left( \left( \frac{1-p_{2}}{p_{2}} \right)\left( \frac{\frac{p_{2}\left( R_{c}\left( U_{3}-U_{4} \right)-\left( C_{3}-C_{4} \right) \right)+R_{c}\left( U_{4}-U_{2} \right)-\left( C_{4}-C_{2} \right)}{p_{2}\left( R_{c}\left( U_{1}-U_{2} \right)-\left( C_{1}-C_{2} \right) \right)}}{1-\frac{p_{2}\left( R_{c}\left( U_{3}-U_{4} \right)-\left( C_{3}-C_{4} \right) \right)+R_{c}\left( U_{4}-U_{2} \right)-\left( C_{4}-C_{2} \right)}{p_{2}\left( R_{c}\left( U_{1}-U_{2} \right)-\left( C_{1}-C_{2} \right) \right)}} \right) \right)-\sum_{i=1}^{n} \omega_{i}\hat{\theta}_{i} \right]$$

1. Explanation of Random effects contours process:

To run this process:

1. We start by defining the limits of trial estimate, trial precision and the number of contour points or “pixels” per row and column of the resulting plot. Contour points refer to the number of individual squares in the grid that are to be coloured. If contour points are set to 𝑁, then there will be a 𝑁x𝑁 grid of $N^{2}$ trials to be evaluated. In other words, the size of the pixels change depending on what number of points, or resolution is chosen.

2. Two nested for-loops systematically run through every combination of trial estimate and trial precision, and in this way the whole new trial outcome space. At each “pixel”, a corresponding trial is added to the meta-analysis, and the decision model is evaluated.

3. A value is tied to that pixel based on which intervention is deemed cost-effective and the pixel is coloured based on that value.

We want to know if the following inequality (like in (S2.4)), is true when the new trial is added to the meta-analysis.

$${NB}_{A}=R_{c}U_{A}-C_{A}> NB_{B}=R_{c}U_{B}-C_{B}$$

Which, by substituting equations (S2.5), is the same as evaluating

| ( | $R_{c}\left( p_{1}U_{1}+\left( 1-p_{1} \right)U_{2} \right)-\left( p_{1}C_{1}+\left( 1-p_{1} \right)C_{2} \right)>R_{c}\left( p_{2}U_{1}+\left( 1-p_{2} \right)U_{2} \right)-(p_{2}C_{1}+\left( 1-p_{2} \right)C_{2})$ |  |
| --- | --- | --- |

We have everything we need to evaluate this except for $p_{1}$ which we can calculate depending on the meta-analysis effect size using equations described in (3.3). After evaluating this we can code the trial according to which intervention is favoured. If it is true (favour new intervention), we will code it as “grey” and if it is false (favour current intervention), we will code it as “white”, to match the colour that it will be shaded as in the plot. After doing this for all $N^{2}$ new trials, a grey coloured square for each trial where the inequality is true is overlayed on a funnel plot of the existing meta-analysis.
